# Acute Sleep Deprivation Is Associated with Altered F-Wave Temporal Dynamics and Nasalis Repetitive Nerve Stimulation Responses: A Paired Electrophysiological Study

**DOI:** 10.3390/brainsci16090955

**Published:** 2026-09-08

**Authors:** Yağmur İnalkaç Gemici, Eylül Ceren Sönmez

**Affiliations:** Department of Neurology, Manisa Celal Bayar University Faculty of Medicine, Manisa 45030, Türkiye

**Keywords:** sleep deprivation, F-wave chronodispersion, repetitive nerve stimulation, nasalis muscle, electrophysiology

## Abstract

**Highlights:**

**What is the main finding?**

- Acute overnight sleep deprivation increased F-wave chronodispersion and reduced persistence, while nasalis RNS showed a modest change that remained within physiological limits.

**What is the implication of the main finding?**

- Acute sleep loss may alter late motor-response variability without clear evidence of clinically relevant neuromuscular transmission failure.

**Abstract:**

Background: Acute sleep deprivation affects cortical excitability and cognition, but its association with spinal motor output and repetitive nerve stimulation (RNS) responses is insufficiently characterized. We evaluated F-wave parameters and low-frequency RNS before and after an overnight on-call period. Methods: In this prospective paired within-participant study, 30 healthy resident physicians underwent standardized electrophysiological assessments before and after an overnight on-call period involving approximately 24 h of self-reported continuous wakefulness. Fatigue was assessed using the Fatigue Impact Scale (FIS). Posterior tibial nerve F-wave stimuli and 3 Hz nasalis RNS stimuli were delivered at each session. Paired parametric or non-parametric tests were selected according to the distribution of within-participant differences, with Benjamini–Hochberg false discovery rate (FDR) correction across electrophysiological comparisons. Results: FIS scores increased after the on-call period. F-wave maximum latency and chronodispersion increased, whereas persistence decreased; minimum latency was unchanged. The signed first-to-fourth compound muscle action potential (CMAP) amplitude change shifted in the decremental direction, while baseline CMAP amplitude remained stable. RNS values at both sessions were within physiological limits. Conclusions: Acute overnight sleep deprivation was associated with altered F-wave temporal organization and a small directional shift in nasalis RNS responses. The RNS finding was statistically significant, but does not constitute evidence of pathological neuromuscular transmission failure. Larger studies with objective sleep monitoring, additional muscles, more F-wave stimuli, and recovery measurements are required.

## 1. Introduction

Sleep is a fundamental physiological process essential for maintaining central nervous system integrity, synaptic plasticity, metabolic homeostasis, and optimal cognitive performance [1,2]. Acute sleep deprivation has been consistently associated with impairments in attention, executive function, reaction time, and decision-making, particularly in populations exposed to prolonged wakefulness, such as healthcare workers [1,3]. Beyond cognitive consequences, increasing evidence suggests that sleep deprivation also modulates neuronal excitability across multiple levels of the nervous system.

The effects of sleep deprivation on cortical excitability have been extensively studied using transcranial magnetic stimulation (TMS), demonstrating increased cortical excitability and reduced intracortical inhibition following prolonged wakefulness [4,5,6]. These findings support the concept that sleep loss shifts the excitation–inhibition balance toward a hyperexcitable cortical state. However, the impact of acute sleep deprivation on the peripheral motor system, including spinal motor neuron excitability and neuromuscular junction (NMJ) transmission, remains insufficiently characterized.

F-wave responses provide a reliable electrophysiological measure of spinal motor neuron excitability and conduction along the entire length of the motor axon [7,8]. Parameters such as F-wave latency, persistence, and chronodispersion reflect the functional state of the motor neuron pool and are widely used in clinical neurophysiology to assess proximal nerve segments and motor neuron excitability. Despite their clinical relevance, data regarding the influence of sleep deprivation on F-wave parameters are scarce and inconclusive.

Repetitive nerve stimulation (RNS) is a well-established method for evaluating neuromuscular transmission, particularly in disorders affecting the NMJ [9,10]. Low-frequency stimulation (2–3 Hz) assesses postsynaptic transmission by analyzing decremental responses in compound muscle action potential (CMAP) amplitudes [11]. However, the potential impact of physiological stressors such as sleep deprivation on NMJ transmission has not been adequately explored in human studies.

Experimental evidence suggests that sleep deprivation may induce structural and functional alterations at the neuromuscular junction. Animal studies have demonstrated synaptic remodeling, altered acetylcholine metabolism, and changes in presynaptic vesicle dynamics following acute sleep deprivation [12]. These findings raise the possibility that even short-term sleep loss may transiently impair neuromuscular transmission in humans.

To date, the combined effects of acute overnight sleep deprivation on F-wave characteristics and facial-muscle repetitive nerve stimulation responses have not been comprehensively investigated in humans. It was hypothesized that acute sleep deprivation would not produce overt peripheral nerve or neuromuscular junction dysfunction in healthy individuals, but might be associated with subtle alterations in peripheral motor-system excitability and neuromuscular response dynamics that remain within conventional physiological limits. Such alterations were expected to be detectable through variability-sensitive F-wave parameters—including latency, persistence, and chronodispersion—together with low-frequency repetitive nerve stimulation, despite preservation of baseline compound muscle action potential amplitude. Accordingly, the present study aimed to compare posterior tibial nerve F-wave parameters and low-frequency (3 Hz) nasalis repetitive nerve stimulation responses before and after an overnight on-call period using a paired within-participant design.

## 2. Materials and Methods

### 2.1. Study Design and Participants

This prospective observational study was conducted at the Electrophysiology Laboratory of the Department of Neurology at a tertiary university hospital. The study protocol was approved by the Ethics Committee of Manisa Celal Bayar University Faculty of Medicine (approval date: 15 April 2026; approval number: 20.478.486/4065), and all procedures were carried out in accordance with the Declaration of Helsinki. Written informed consent was obtained from all participants prior to inclusion.

A total of 30 resident physicians were enrolled. Inclusion and exclusion criteria were applied to minimize potential confounding factors that could affect peripheral nerve conduction or neuromuscular transmission.

### 2.2. Inclusion and Exclusion Criteria

Adult resident physicians who were actively working overnight on-call shifts were eligible. Before the baseline assessment, participants reported at least 5 h of sleep; mean reported sleep duration was 6.61 ± 1.01 h. They were instructed not to consume caffeine, tea, energy drinks, or other stimulants during the 2 h preceding either electrophysiological assessment. Participants reported remaining awake throughout the overnight duty until completion of the post-call assessment. Written informed consent was obtained before enrollment.

Participants with peripheral neuropathy, radiculopathy, plexopathy, motor neuron disease, myopathy, neuromuscular junction disease, or another neurological condition that could influence the measurements were excluded. Exclusion criteria also included diabetes mellitus, thyroid dysfunction, renal or hepatic disease, electrolyte imbalance, vitamin B12 deficiency, diagnosed sleep disorders, recent acute illness, and previous surgery involving the examined facial or tibial nerve. No participant had a known neurological or systemic disease or used medication capable of affecting peripheral nerve conduction, motor neuron excitability, or neuromuscular transmission.

### 2.3. Study Protocol

Each participant underwent two electrophysiological evaluations at approximately 09:00: a baseline assessment before the on-call period, and a post-call assessment the following morning after approximately 24 h of self-reported continuous wakefulness. Testing at the same time was chosen to reduce time-of-day and circadian-phase differences between sessions. All participants worked in the same department under broadly comparable on-call conditions and workload. Sleep duration before baseline and wakefulness during the on-call period were recorded only by self-report; neither sleep deprivation nor possible brief sleep episodes were objectively verified by polysomnography or actigraphy. Subjective fatigue was assessed at both sessions using the FIS. Accordingly, this observational protocol evaluates associations with an overnight on-call period and cannot isolate sleep loss from workload, stress, or other session-dependent exposures.

### 2.4. Fatigue Assessment

Fatigue severity was evaluated using the Fatigue Impact Scale (FIS), a validated instrument assessing the effects of fatigue on cognitive, physical, and psychosocial functioning [13,14]. The questionnaire was administered in a standardized manner by the same investigator (E.C.S.) before and after the on-call period. Participants completed the scale in a quiet environment, and clarification was provided when necessary to ensure accurate understanding of individual items. Total and subscale FIS scores were recorded for all participants. To ensure methodological consistency, the same version of the questionnaire and identical administration procedures were used at both assessment time points.

### 2.5. Electrophysiological Assessments

All electrophysiological studies were performed using the same Electromyography (EMG) device (Dantec Keypoint Focus EMG system (Natus Medical Incorporated, Middleton, WI, USA) under standardized laboratory conditions. Skin temperature was maintained above 32 °C, and ambient room temperature was kept constant between 22 °C and 24 °C throughout all recordings.

To reduce inter-operator variability and improve reproducibility, all examinations were performed by the same experienced operator using identical EMG equipment and acquisition settings for all participants. Electrode placement was standardized according to anatomical landmarks [15,16] and additionally documented using photographic references to ensure consistent positioning between pre-call and post-call recordings. Stimulation intensity was adjusted to supramaximal levels and maintained consistently within each participant across both sessions. Each participant served as their own control, and all measurements were repeated under identical technical conditions before and after sleep deprivation.

Care was taken to minimize recording artifacts by ensuring low skin impedance, stable electrode contact, participant relaxation during recordings, and avoidance of excessive movement during the stimulation and acquisition procedures.

### 2.6. F-Wave Study

F-wave recordings were obtained from the posterior tibial nerve using standard surface electrodes, with the active electrode placed over the abductor hallucis muscle and the reference electrode positioned distally [8]. Supramaximal electrical stimulation was delivered at the ankle. Recordings were performed using identical filter settings, sweep speed, and sensitivity parameters across sessions (sweep speed: 8 ms/division; sensitivity: 5 mV/division for M-wave recordings and 1 mV/division for F-wave recordings).

Exactly 10 supramaximal stimuli were delivered to every participant in both sessions. Minimum and maximum F-wave latencies, chronodispersion, and persistence were analyzed. Chronodispersion was defined as the difference between the latest and earliest detectable F-wave latencies in the 10-stimulus series. Persistence was calculated as the proportion of the 10 stimuli that elicited a detectable F-wave. Maximum latency was evaluated as a sampling-dependent descriptor of the latest recurrent response and was interpreted only together with chronodispersion and persistence, not as a stand-alone routine diagnostic marker. This stimulus count was consistent with the standardized protocol used to establish the laboratory-specific reference values and is within the range used for routine F-wave assessment in healthy individuals. Although the paired design, identical stimulus count, and standardized recording conditions improved within-participant comparability, no validated minimum number of stimuli was established for reliable test–retest measurement of tibial F-wave chronodispersion or persistence in healthy individuals over a 24 h interval. Moreover, chronodispersion is inherently sensitive to the sampling of extreme latencies, and persistence has a resolution of 10 percentage points when based on 10 stimuli. Therefore, the findings were interpreted as group-level paired changes rather than evidence of reliable individual-level change or diagnostic abnormality.

### 2.7. Repetitive Nerve Stimulation (RNS)

Low-frequency (3 Hz) RNS was performed only on the nasalis muscle using surface electrodes, with the active electrode positioned over the nasalis and the reference electrode over the nasal dorsum [15,16,17,18,19,20]. The facial nerve was stimulated at a site producing a stable baseline CMAP, and intensity was adjusted to supramaximal levels. The nasalis was selected because it is a clinically established facial RNS recording site and can provide a cleaner biphasic CMAP with fewer stimulus artifacts than the orbicularis oculi; prior technical work met predefined recording-quality criteria more consistently in the nasalis than in the orbicularis oculi [18]. Studies in myasthenia gravis also support the diagnostic sensitivity of the nasalis among facial muscles, particularly in bulbar-predominant disease [19,20]. This selection was intended to maximize within-participant recording stability, not to imply that findings from one facial muscle generalize to limb muscles or other facial muscles. No limb muscle or other facial muscle was examined.

Ten sequential CMAP responses were recorded during each 3 Hz train. Baseline CMAP amplitude and the signed percentage change from the first to the fourth CMAP were analyzed. The signed change was calculated as [(CMAP4 − CMAP1)/CMAP1] × 100. Thus, positive values represent an increment, whereas negative values represent a decrement. This variable is reported as signed first-to-fourth CMAP amplitude change rather than as “decrement percentage” to avoid ambiguity. Identical sweep speed, sensitivity, filter settings, electrode montage, positioning strategy, and stimulation protocol were used at both sessions.

All electrophysiological data were recorded digitally and stored in a coded format. Data entry was performed by the same investigator and independently verified by a senior neurophysiologist to minimize recording errors.

### 2.8. Statistical Analysis

Statistical analyses were performed using IBM SPSS Statistics, Version 25.0 (IBM Corp., Armonk, NY, USA), with independent verification of the revised analyses. Normality was evaluated for within-participant differences using histograms, Q–Q plots, and the Shapiro–Wilk test. Normally distributed paired differences were analyzed using paired-samples *t*-tests; non-normally distributed differences were analyzed using Wilcoxon’s signed-rank tests. For transparency, non-parametric sensitivity analyses were also performed for all paired electrophysiological comparisons and produced the same pattern of statistical significance. Benjamini–Hochberg false discovery rate correction was applied across the electrophysiological comparisons (F-wave minimum latency, maximum latency, persistence, chronodispersion, baseline RNS CMAP amplitude, and signed first-to-fourth CMAP change). The FIS was analyzed separately as the subjective fatigue outcome. Both raw and FDR-adjusted *p* values are reported; two-sided adjusted *p* < 0.05 was considered significant. Continuous data are presented as mean ± standard deviation to facilitate comparison with the prior electrophysiological literature, and mean paired differences with 95% confidence intervals are provided for normally distributed differences.

## 3. Results

**Figure 1:**
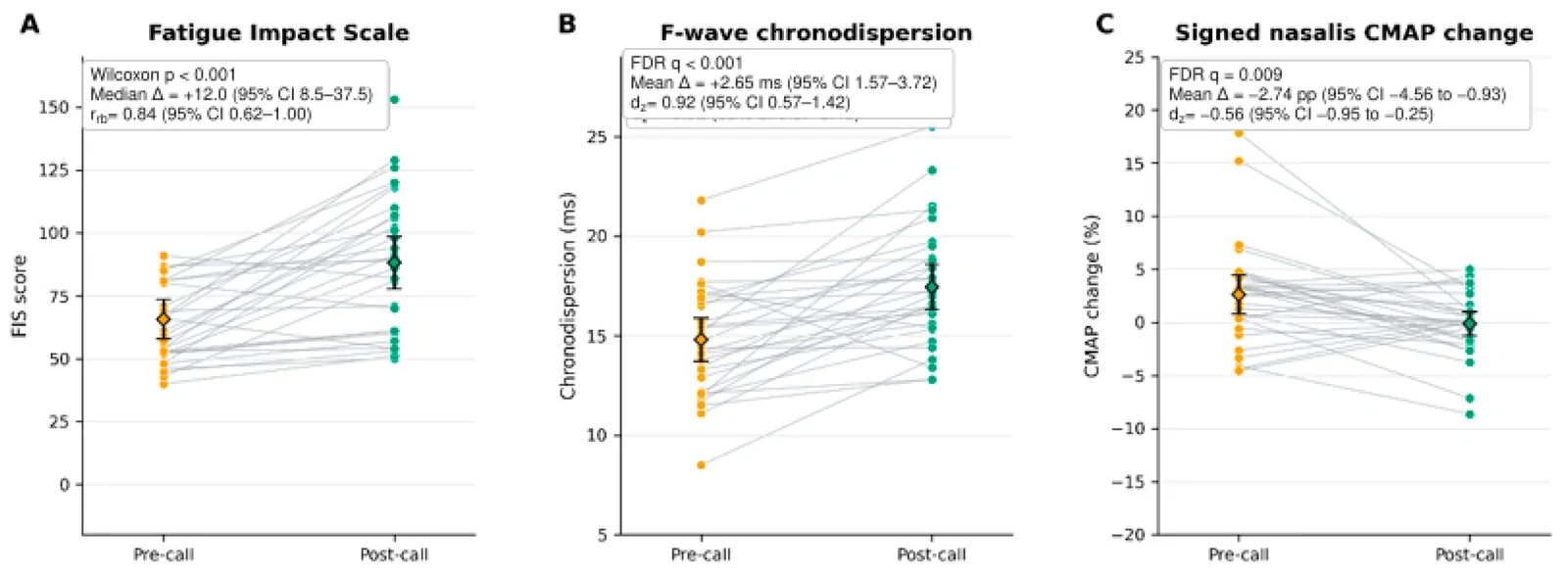
Quantified paired changes after acute sleep deprivation: Panel (**A**) shows the increase in FIS score after the on-call period. Panel (**B**) shows the increase in F-wave chronodispersion. Panel (**C**) shows the paired shift in signed first-to-fourth nasalis CMAP amplitude change. Positive values denote an increment, and negative values denote a decrement. The RNS values remained within physiological limits. Colored markers indicate individual observations, and gray lines connect the paired pre-call and post-call values of the same participant; black diamonds and error bars indicate the group-level summary estimates and their 95% confidence intervals; statistical comparisons are paired and two-sided. FIS: Fatigue Impact Scale; CMAP: compound muscle action potential.

Thirty resident physicians (13 males and 17 females; mean age: 29.77 ± 5.28 years; range: 24–51 years) were included. Mean height was 1.73 ± 0.09 m, mean BMI was 24.08 ± 3.91 kg/m^2^, and mean self-reported sleep duration before baseline was 6.61 ± 1.01 h. All baseline F-wave latency, chronodispersion, and persistence measurements were within the laboratory reference limits. After the overnight on-call period, FIS scores increased from 65.70 ± 20.94 to 88.13 ± 27.65 (Wilcoxon’s *p* < 0.001; Figure 1A).

F-wave maximum latency increased from 62.73 ± 5.41 to 65.73 ± 5.80 ms (mean paired difference 3.00 ms, 95% CI 1.80–4.20; raw *p* < 0.001; FDR-adjusted *p* < 0.001), and chronodispersion increased from 14.80 ± 2.91 to 17.45 ± 3.03 ms (raw *p* < 0.001; FDR-adjusted *p* < 0.001; Figure 1B). Persistence decreased from 94.3 ± 11.4% to 85.0 ± 20.1% (Wilcoxon’s raw *p* = 0.012; FDR-adjusted *p* = 0.018). Minimum latency did not change significantly (raw *p* = 0.119; FDR-adjusted *p* = 0.143).

Nasalis RNS showed a directional shift in the signed first-to-fourth CMAP amplitude change, from a small mean increment before the on-call period (+2.62 ± 4.92%) to an essentially unchanged/slightly decremental mean response afterward (−0.12 ± 3.00%). The mean paired shift was −2.74 percentage points (95% CI −4.56 to −0.93; raw *p* = 0.004; FDR-adjusted *p* = 0.009; Figure 1C). Baseline CMAP amplitude remained stable (2.25 ± 1.20 vs. 2.22 ± 0.95 mV; Wilcoxon’s raw and FDR-adjusted *p* = 0.593). Importantly, RNS responses at both time points remained within accepted physiological limits.

All electrophysiological findings are summarized in Table 1.

**Figure 2:**
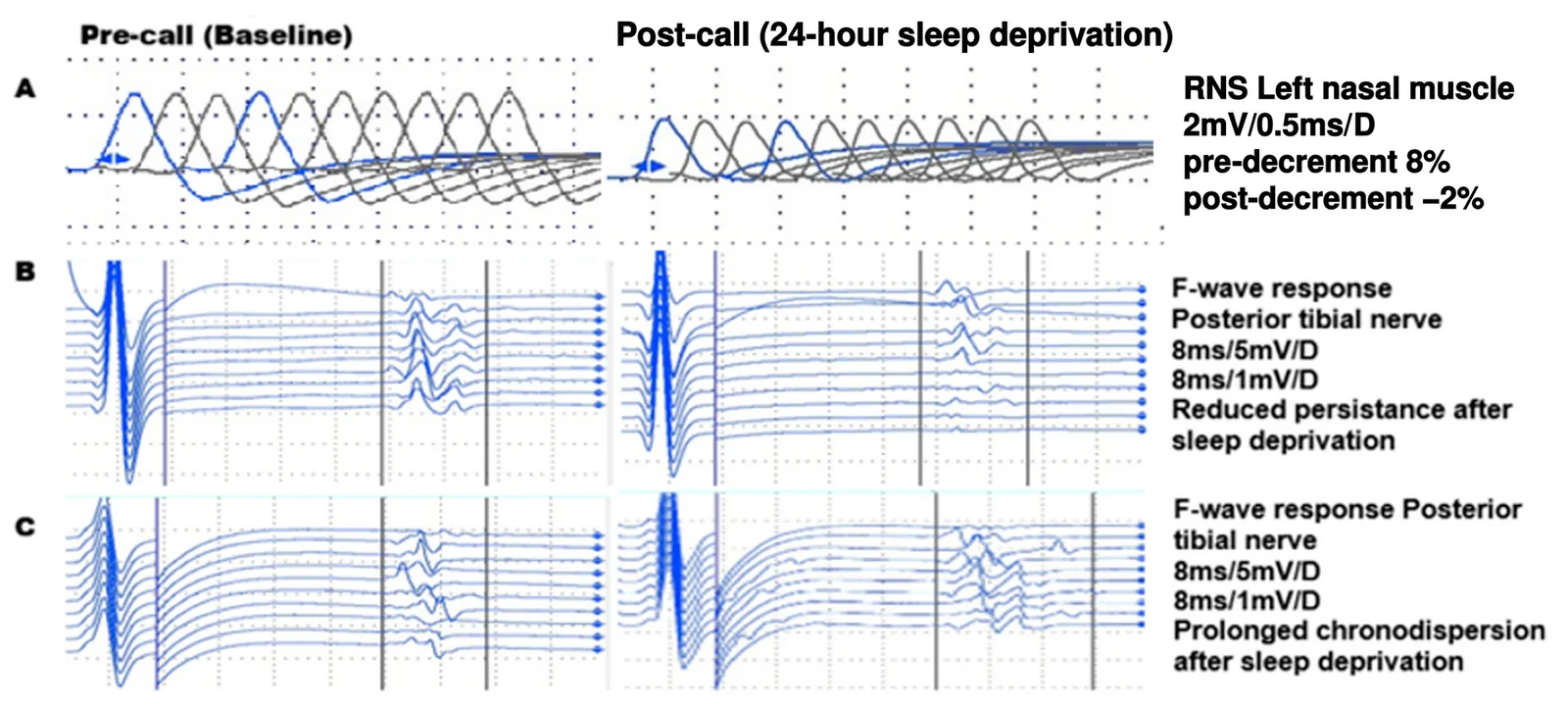
Representative electrophysiological recordings before and after the overnight on-call period: (**A**) Nasalis RNS recordings, displayed at 2 mV/division and 0.5 ms/division, illustrate measurement of the signed first-to-fourth CMAP amplitude change and should not be interpreted as pathological decrement. (**B**) Posterior tibial F-wave recordings, displayed at 5 mV/division for the M response, 1 mV/division for F-waves, and 8 ms/division, illustrate reduced sampled persistence. (**C**) Recordings with the same scales illustrate broader temporal dispersion of sampled F-waves. Pre-call and post-call traces were acquired using identical filter settings and supramaximal stimulation strategy.

Representative recordings are presented in Figure 2 using identical gain, sweep speed, filter settings, and stimulation strategy. The examples illustrate the signed first-to-fourth CMAP measurement and the broader temporal spread of F-waves after the on-call period; they are illustrative and are not intended to imply pathological RNS decrement.

## 4. Discussion

In this prospective paired within-participant study, subjective fatigue increased after an overnight on-call period, F-wave maximum latency and chronodispersion increased, and persistence decreased, whereas minimum latency and baseline CMAP amplitude remained stable. A small but statistically significant shift in signed nasalis RNS response occurred in the decremental direction, but the group mean remained essentially unchanged and well within physiological limits. Accordingly, the principal finding is altered temporal organization of sampled F-wave responses; the RNS result should be regarded as an exploratory, subclinical directional change rather than evidence of neuromuscular junction dysfunction.

The recent indexed human literature on acute sleep deprivation has focused predominantly on cortical physiology, and those data are directly relevant to interpreting our F-wave results [6,21]. In a constant-routine TMS-EEG study, Ly et al. [6] showed that cortical excitability is jointly shaped by time awake and circadian phase, emphasizing that prolonged wakefulness itself measurably perturbs the excitation–inhibition balance. Salehinejad et al. [21] subsequently reported that overnight sleep deprivation upscales cortical excitability, enhances glutamatergic facilitation, reduces or reverses GABAergic inhibition, and alters plasticity-related responses. However, a more recent threshold-tracking TMS study found relatively little change across several cortical excitability measures after 24 h of sleep deprivation [22], apart from short-latency afferent inhibition, and a 2025 meta-analysis concluded that the overall direction of evidence favors reduced inhibition and enhanced excitability but remains heterogeneous across paradigms [23]. Taken together, the human TMS literature does not support a single, uniform “hyperexcitable” state; rather, it points to a reweighting of inhibitory, facilitatory, and sensory-gated inputs under sleep loss.

Direct human evidence at the spinal or peripheral motor-output level is much sparser, but the available studies are strikingly compatible with our pattern. Gonçalves et al. [24] found that 24 h sleep deprivation preserved H-reflex and V-wave responses, suggesting that coarse spinal/Ia circuitry and descending drive can remain intact after a single night awake. In a related study, the same group found no meaningful change in soleus EMG amplitude or explosive torque production, implying that gross motor output metrics may be relatively insensitive to acute sleep loss [25]. Yet Oliveira et al. [26] showed that one night of sleep deprivation increased the regularity of isometric torque fluctuations, indicating loss of adaptability in motor output despite preserved peak force metrics. Rault et al. [27] also demonstrated that one night of sleep deprivation reduces respiratory motor output, primarily through its cortical component. This dissociation between preserved gross amplitude–based measures and impaired temporal organization is closely aligned with our data: minimum F-wave latency and baseline CMAP amplitude were preserved, whereas variability-sensitive late-response metrics changed significantly.

The F-wave findings are most appropriately interpreted as altered temporal variability rather than a uniform change in spinal motor neuron excitability. Preserved minimum latency suggests stability of the fastest-conducting motor axons, whereas increased maximum latency and chronodispersion indicate a wider range of recurrent-response timing among the sampled responses. Lower persistence indicates that fewer of the 10 stimuli elicited a recordable F response, but it does not demonstrate temporary inactivation or loss of motor neurons. Persistence is influenced by motor-neuron-pool excitability, recurrent response probability, technical detection, and the number of stimuli sampled. Because only 10 stimuli were delivered, maximum latency may have undersampled rare late responses, and both chronodispersion and persistence have appreciable finite-sampling variability. The concordant direction of these measures is noteworthy, but it does not remove this limitation. Recent work suggesting that F-waves may involve spinal microcircuits activated through recurrent motor-axon collaterals provides a possible framework for state-dependent changes in temporal organization [28], although the present design cannot determine the underlying mechanism.

All participants were young, healthy physicians without known neurological disease or clinically apparent motor impairment. Therefore, the observed reduction in F-wave persistence should not be interpreted as temporary motor-neuron inactivation or clinically relevant motor dysfunction. Rather, it may reflect a subtle, physiological-range variation in the probability of recurrent motor-neuron discharge within the limited number of recorded responses. Because objective motor-performance testing was not included and F-waves were recorded from the posterior tibial nerve, no direct conclusions can be drawn regarding fine motor function. Further studies would be required to determine whether these subtle electrophysiological changes have any measurable functional correlate.

The RNS result also warrants a balanced interpretation. The signed first-to-fourth CMAP change shifted from a small increment before the on-call period to a slightly decremental response afterward. This paired difference remained significant after false discovery rate correction and in Wilcoxon’s sensitivity analysis. Because each participant served as their own control and both examinations used the same protocol, the result is not readily explained by stable interindividual characteristics. However, the paired design cannot exclude session-dependent technical or physiological variability. Nevertheless, because wakefulness was self-reported and there was no rested repeated-measures control condition, the change cannot be attributed causally to sleep deprivation and may also reflect workload, stress, unmeasured stimulant exposure, or session-dependent technical and physiological variability. The absolute responses remained within accepted physiological limits; therefore, the finding represents a subtle association with the post-call state, not evidence of pathological neuromuscular transmission failure.

The nasalis was selected because facial CMAP quality criteria can be achieved more consistently in this muscle than in the orbicularis oculi [18]. It is also an established RNS recording site in myasthenia gravis, with reported sensitivity in bulbar disease and performance comparable to other facial muscles in some cohorts [19,20]. Nevertheless, facial RNS remains susceptible to electrode position, volume conduction, movement, and waveform morphology [29]. Examination of the nasalis alone therefore limits generalization of the RNS finding to other muscles.

Several mechanisms may contribute to these observations. Experimental studies indicate that acute sleep deprivation can affect neuromuscular junction structure, muscle metabolism, and oxidative balance [12,30,31]. Human studies also describe sleep loss–related changes in adenosinergic signaling, autonomic regulation, stress responses, and inflammatory pathways, although endocrine and inflammatory findings are heterogeneous [32,33,34,35,36,37,38]. These mechanisms provide biological plausibility for a transient motor-system effect but cannot be distinguished by the present design.

The clinical implications should, therefore, remain limited. Sleep deprivation did not produce pathological RNS decrement or a pattern diagnostic of peripheral neuropathy. Nonetheless, recent sleep status may be relevant when small or borderline changes are encountered in variability-sensitive F-wave measures or technically demanding facial RNS recordings. Reassessment after adequate sleep may be considered when an equivocal electrophysiological finding is inconsistent with the clinical picture, although this study does not define a diagnostic threshold or false-positive rate.

The paired within-subject design, testing at the same time, with same-operator recordings, temperature control, standardized electrode placement, fixed stimulus counts, and recruitment from a single department support internal comparability. Several limitations require emphasis. First, wakefulness and baseline sleep duration were self-reported; without actigraphy or polysomnography, brief sleep episodes, baseline partial sleep restriction, and interindividual differences in sleep debt cannot be excluded. Second, the absence of a rested repeated-measures control condition prevents separation of sleep loss from test–retest variability, overnight workload, stress, circadian influences not eliminated by clock-time matching, or other on-call exposures. Workload, stress, and stimulant exposure were not objectively quantified or biochemically verified. Third, only 10 F-wave stimuli were acquired. Although this fixed protocol matched our laboratory reference procedure and was identical across paired sessions, it provides limited resolution for persistence and may undersample extreme latencies; therefore, the observed changes in maximum latency and chronodispersion should be considered sampling-sensitive and require confirmation with larger stimulus trains. Resampling the same 10 acquired responses cannot recover late responses that were never sampled and, therefore, cannot fully resolve this acquisition limitation. Fourth, only the nasalis muscle was examined, limiting generalization to other facial muscles and limb muscles; small facial RNS changes remain susceptible to electrode position, volume conduction, movement, and waveform morphology. Finally, the modest sample comprised healthy resident physicians from a single department, most of whom were young adults; the findings may not generalize to older adults, patients with neurological or sleep disorders, different occupational settings, repeated or chronic sleep restriction, or clinically relevant electrodiagnostic abnormalities.

## 5. Conclusions

In conclusion, an overnight on-call period was associated with altered F-wave temporal dynamics and a modest, paired shift in nasalis RNS response despite preserved minimum latency, baseline CMAP amplitude, and physiological-range RNS values. These results support a transient effect on motor-system response dynamics rather than clinically manifest peripheral nerve or neuromuscular junction dysfunction. Replication with objective sleep monitoring, larger F-wave samples, multiple RNS muscles, rested control sessions, and recovery-sleep assessment is needed.

## Figures and Tables

**Table 1:** Clinical and electrophysiological findings before and after 24 h sleep deprivation.

Variable	Pre-Call Mean ± SD	Post-Call Mean ± SD	Raw *p*	FDR q
**Age**	29.77 ± 5.28			
**Sex (M/F)**	13/17			
**Height (m)**	1.73 ± 0.09			
**BMI**	24.08 ± 3.91			
**Last sleep duration (hours)**	6.61 ± 1.01			
**FIS**	65.70 ± 20.94	88.13 ± 27.65	<0.001	—
**F-wave minimum latency (ms)**	47.77 ± 4.31	48.33 ± 4.29	0.119	0.143
**F-wave maximum latency (ms)**	62.73 ± 5.41	65.73 ± 5.80	<0.001	<0.001
**F-wave persistence (%)**	94.3 ± 11.4	85.0 ± 20.1	0.012	0.018
**F-wave chronodispersion (ms)**	14.80 ± 2.91	17.45 ± 3.03	<0.001	<0.001
**Repetitive CMAP amplitude (mV)**	2.25 ± 1.20	2.22 ± 0.95	0.593	0.593
**Signed first-to-fourth CMAP amplitude change (%)**	2.62 ± 4.92	−0.12 ± 3.00	0.004	0.009

Raw *p* values derive from paired-samples *t*-tests when within-participant differences were normally distributed and Wilcoxon’s signed-rank tests otherwise. Benjamini–Hochberg FDR correction was applied across six electrophysiological comparisons; FIS was analyzed separately. Positive signed CMAP change denotes an increment and negative change denotes a decrement. M: male; F: female; BMI: body mass index; FIS: Fatigue Impact Scale; CMAP: compound muscle action potential.

## Data Availability

The de-identified participant-level dataset supporting the findings of this study are available from the corresponding author upon reasonable request. The data are not publicly available due to privacy and ethical considerations related to participant-level clinical and electrophysiological data.
